# Schistosomiasis in Western Lake Turkana, Kenya: An Exploratory Serosurvey and Validation of Dried Blood Spots for Field Studies

**DOI:** 10.3390/tropicalmed11040091

**Published:** 2026-03-30

**Authors:** Andrea Miján, Oihane Martín, Esther Ciancas, Carmen Llorente Martín, Gilechrist Lokoel, Sarah Lokaala, Daniel Lokiriama, Sagrario de la Fuente Hernanz, María Llorente de Santiago, Ana Camila Bertomeu, Jose A. Perez-Molina

**Affiliations:** 1 Microbiology Department, Hospital Universitario de Getafe, Carretera Madrid-Toledo km 12,500, 28905 Getafe, Spain; andrea.mijan.perez@gmail.com; 2 Microbiology Department, Hospital Universitario Ramón y Cajal (IRYCIS), Carretera de Colmenar km 9100, 28034 Madrid, Spain; oihane.martin.s@gmail.com (O.M.); sagrariopedrezuela5@gmail.com (S.d.l.F.H.); camibertomeu@gmail.com (A.C.B.); 3 CIBER de Enfermedades Infecciosas (CIBERINFEC), Instituto de Salud Carlos III, Avenida de Monforte de Lemos 5, 28029 Madrid, Spain; 4 Ophthalmology Department, Hospital Universitario Ramón y Cajal (IRYCIS), Carretera de Colmenar km 9100, 28034 Madrid, Spain; esther.ciancas@salud.madrid.org; 5 Primary Care Health Center Collado Villalba Estación, Calle Los Madroños, 5, 28400 Collado Villalba, Spain; mamenllorente@hotmail.com; 6 Turkana County Medical Services, Turkana County Government, Lodwar P.O. Box 11-30500, Kenya; lockhell80@gmail.com; 7 Policy Planning, Monitoring and Evaluation, Turkana County Government, Lodwar P.O. Box 11-30500, Kenya; sarahlokaala@yahoo.com; 8 Turkana Medical Services, Turkana North Sub-County, Turkana County Government, Lodwar P.O. Box 11-30500, Kenya; danielechor@yahoo.com; 9 Lobur Mobile Clinic, Fundación Emailakat, Calle Bocángel 28, 3°dcha, 28028 Madrid, Spain; maria.llorentedesantiago@gmail.com; 10 National Reference Centre for Imported Tropical Diseases, Infectious Diseases Department, Hospital Universitario Ramón y Cajal (IRYCIS), Carretera de Colmenar km 9100, 28034 Madrid, Spain

**Keywords:** schistosomiasis, Kenya, dried blood spot, screening, epidemiology, diagnosis

## Abstract

Background: Schistosomiasis remains a significant neglected tropical disease in Kenya, but its presence in the western/northern Lake Turkana region is poorly characterised. We conducted an exploratory serosurvey to assess evidence of *Schistosoma* spp. exposure and a diagnostic accuracy study to evaluate dried blood spots (DBSs) for field serology. Methods: We performed a cross-sectional survey in adults (≥18 years) from six communities in the western/northern and shoreline area of Turkana Lake, excluding individuals with >6 months of residence in other Kenyan endemic areas. Capillary blood was collected on DBSs and tested centrally using ELISA for *Schistosoma* spp. IgG. In parallel, DBS cards performance was compared with paired routine serum ELISA in 23 patients assessed for suspected schistosomiasis at our centre. Results: We enrolled 155 participants (60% men; median age 30 years), with nearly universal reported freshwater contact (154/155, 99.4%). In the validation study, DBS values were lower than serum values (mean bias 0.27), with moderate correlation (*r* = 0.54) and modest discrimination (AUC 0.65; sensitivity 80% and specificity 50% at OD index >0.8). The median DBS ELISA OD index for the 155 participants was 0.55 (IQR, 0.34–0.79). Six samples exceeded 0.8, but these values were low, and all had negative IHA (<1/80), yielding no confirmed seropositive cases. Conclusions: These findings suggest low or absent sustained transmission in the sampled communities during the study period and indicate that DBS-based serology is operationally feasible but requires careful calibration and confirmatory testing for robust field inference.

## 1. Introduction

Schistosomiasis affects millions of people, especially in Sub-Saharan Africa, and causes serious health problems. Although it has been studied in Kenya, little is known about its presence in the western region of Lake Turkana. There are indications that active foci of the disease may exist in that area. Consequently, a seroprevalence study was conducted to detect specific antibodies and evaluate active the transmission of the parasite. We also carried out a preliminary diagnostic accuracy study to validate dried blood spot (DBS) sampling for serological testing, a practical approach for field studies in resource-limited settings.

Schistosomiasis is among the most common parasitic tropical diseases worldwide. In 2021, more than 251 million individuals were infected, spanning 70 countries, with 90% of cases concentrated in Sub-Saharan Africa. The WHO classifies it as a neglected tropical disease (NTD) with considerable health and social impacts on populations with limited socioeconomic resources [1,2,3]. The species mainly responsible for causing schistosomiasis in humans are *Schistosoma haematobium*, which leads to urogenital involvement and is found across Sub-Saharan Africa and the Middle East, and *S. mansoni*, which is also present in parts of the Caribbean and South America (Brazil, Suriname, and Venezuela), causing intestinal and hepatic involvement. Other less common species that cause hepatointestinal involvement include the following: *S. japonicum*, mainly found in the Philippines and China, *S. mekongi*, limited to the Mekong River basin and *S. guineensis* and *S. intercalatum*, in regions of the central African tropical rainforest [2,4].

This parasitosis is acquired through contact with freshwater contaminated with cercariae, with infection occurring when larvae penetrate the skin. In endemic areas, repeated exposure from a young age often leads to a chronic condition. Although up to 60% of infections are asymptomatic and diagnosed through screening, retained eggs can cause granulomatous inflammation and significant morbidity, progressing from an active, treatable stage to chronic disease where damage may become irreversible [5,6]. Clinical signs are often nonspecific (e.g., abdominal pain, diarrhoea, fatigue, haematuria), but severe outcomes include portal hypertension, hepatosplenomegaly, bladder cancer, and neurological or pulmonary conditions [2,7]. In addition, in women, urogenital schistosomiasis is linked to chronic symptoms such as dysuria, abnormal vaginal discharge or bleeding, and reproductive health issues [8,9].

In Kenya, schistosomiasis is an endemic parasitic disease, with an estimated 4.23 million people needing preventive treatment annually, of whom just over half are school-aged children (https://apps.who.int/neglected_diseases/ntddata/sch/sch.html, accessed on 4 February 2026). The geographical distribution of schistosomiasis in Kenya indicates that infection with *S. mansoni* is more common in the Lake Victoria basin and the central highlands [10]. In contrast, infection with *S. haematobium* is prevalent along the Kenyan coast, in the Tana River, and in Lake Victoria. Recent studies report a national prevalence of schistosomiasis of 5.0% (95% CI: 4.9–5.2%) [10]. Specifically, *S. mansoni* has a prevalence of 3.0% (95% CI: 2.9–3.1%), while *S. haematobium* accounts for 2.2% (95% CI: 2.1–2.3%) [11]. The prevalence varies significantly across different regions in Kenya. For example, in Tana River County, *Schistosoma haematobium* prevalence has been reported at 19.6% (95% CI: 11.6–31.3%) among school-aged children [12]. In areas near Lake Victoria, such as Mbita, the prevalence of *S. mansoni* can reach up to 45.1% (95% CI: 41.7–48.5%) [13]. Mass treatment programs have significantly reduced the prevalence of infection, especially among school-aged children [11]. However, there have been few studies on the prevalence of this infection in the Turkana region. Therefore, the disease burden in this area, although likely low, remains uncertain [10].

For diagnosis, microscopy-based methods depend on egg excretion and may fail to detect infections during the acute phase or when egg excretion is low. Methods such as Kato–Katz or FLOTAC can enhance sensitivity. [14]. PCR increases sensitivity and can detect early infection, but it is less useful for post-treatment follow-up because parasite DNA may persist for months to a year, and access remains limited [14,15]. Consequently, serology is most widely used: assays such as ELISA, indirect haemagglutination, and immunofluorescence provide good sensitivity and specificity—often improved by combining tests—and can detect infection even before egg output, although they cannot reliably differentiate species or distinguish between active and past infection [14,16,17]. Moreover, in remote or resource-limited settings, DBSs may offer a practical alternative for sample collection in schistosomiasis field surveys [18,19].

We aimed to estimate schistosomiasis seroprevalence by detecting *Schistosoma*-specific antibodies using DBS sampling in a representative population from communities in western Lake Turkana and along its shoreline. DBS specimens were analysed in a centralised laboratory in Spain, and the approach was validated against paired serum samples from individuals with known positive and negative schistosomiasis serology.

## 2. Material and Methods

### 2.1. Study Setting and Participant Recruitment

This is an observational, cross-sectional study of adults (≥18 years) residing in the western and northern parts of Lake Turkana County, Kenya.

Participants were recruited by convenience sampling from communities in the study area where local contact had been established and fieldwork was feasible. Individuals with residence periods longer than six months in known endemic areas of the country or with a potential risk of exposure to *Schistosoma* spp. were excluded. We aimed to detect the presence of antibodies against *Schistosoma* spp. among the local population of the Lake Turkana region in order to determine whether this parasitic disease is present in the northern part of the county and to treat affected individuals. Samples were collected in several locations:

- Lobur Mission, where several cases of expatriates with positive serology have been observed at our clinic in Ramón y Cajal University Hospital. The main risk factor seemed to be a nearby dam.
- Todonyang Mission, where there is a community of fishermen on the shores of Lake Turkana.
- Loropio, in the Lokitoengaber Area, where an NGO has a project with fishermen.
- Nariokotome Dispensary, near Lake Turkana.

We met with local representatives several months before starting fieldwork to introduce and explain the project to community members. Once the fieldwork began, we visited previously designated sites—usually local health centres—where people gathered for screening. In cases where the population was difficult to reach, we directly visited their villages to ensure that everyone had access to the screening process. Our team consisted of a mediator–translator, healthcare professionals, and logistics staff.

The study variables were recorded on a paper form designed for this study, which the investigators kept. The information was then transferred to a computerised database for anonymisation before further analysis. The variables included socio-demographic characteristics, usual residence region, freshwater baths (dams, ponds, or Turkana Lake), and analytical results.

### 2.2. Sample Collection and Laboratory Procedures

For blood sampling during fieldwork, we used filter paper (DBS Tropbio^®^ filter paper disk for blood collection, Cellabs, Sydney, Australia). Blood samples were obtained by pricking the finger and touching each of the six circular extensions on the disk to a droplet of whole blood. Each extension absorbs approximately 10 μL of blood. The filters were dried at room temperature for at least two hours and then stored in sealed plastic envelopes; once they were transported to in the central laboratory, they were stored at −80 °C until processing.

Two filter paper circles were placed in a 2.0 mL microfuge tube, and 120 µL of saline solution was added to achieve a 1:10 concentration (assuming a 40% hematocrit as a correction factor). The filter paper remained immersed overnight at 4 °C. On the following day, the contents were gently mixed using a vortex and centrifuged at 10,000× *g* for 15 min. Finally, the supernatant was carefully collected with a pipette for detecting antibodies against *Schistosoma* spp. by ELISA.

Regarding serological diagnosis, two commercial tests were combined to detect IgG antibodies against *Schistosoma* spp.: an ELISA (*Schistosoma mansoni* IgG NovaLisa^®^, NovaTec Immunodiagnostica, GMBH, Waldstrasse 23 A6, 63128 Dietzenbach, Germany) and an indirect haemagglutination assay (SCHISTOSOMIASIS FUMOUZE^®^, ELITech Group, 13-15 rue Jean Jaurès—92800 Puteaux, France). A result was considered positive if the ELISA OD index exceeded 1.1 or the IHA titre was ≥1/160 (with 1/80 treated as indeterminate).

To evaluate DBS performance against routine serology, we recruited 23 patients at our centre who were assessed for suspected schistosomiasis and underwent standard diagnostic testing as part of clinical care. During the same visit, an additional capillary blood sample was collected on DBS cards and processed later on, as described above. DBS eluates were tested for anti-*Schistosoma* IgG, and the resulting indices were compared with the corresponding routine serological results obtained from venous samples (ELISA testing).

### 2.3. Statistical Analysis

Categorical data are presented as absolute numbers and proportions, while continuous variables are expressed as medians and interquartile ranges (IQRs). The χ^2^ test or Fisher’s exact test was used to compare categorical variables, and Student’s *t*-test or ANOVA was employed for continuous variables. Data processing and analysis will be conducted using the statistical package STATA v. 18.0. (StataCorp LP, College Station, TX, USA).

Assuming that the prevalence of antibodies against *Schistosoma* spp. was 10% in the study population, indicating either active or past infection, we estimated that screening 154 individuals would be required to detect at least 10 seropositive patients (with a 95% confidence).

Agreement between the ELISA index and the DBS-derived index was evaluated using complementary concordance methods. We first quantified the linear association with Pearson’s correlation coefficient and then estimated overall agreement with Lin’s concordance correlation coefficient (CCC), which combines precision (correlation) and accuracy (deviation from the 45° line of identity). To further characterise agreement across the range of measurements, we performed a Bland–Altman analysis by calculating the mean difference (ELISA − DBS) and the 95% limits of agreement (mean difference ± 1.96 SD), and we assessed proportional bias by correlating the paired differences with their means. In addition, receiver operating characteristic (ROC) curves were used to compare the diagnostic accuracy of DBSs against the reference standard, summarised by the area under the curve (AUC).

### 2.4. Ethics Statement

This study was approved by the Ethics Committee for Medicinal Products at Ramón y Cajal University Hospital (approval code: 066/24, approval date: 25 April 2024). The information gathered in this study was treated as confidential and handled accordingly at all times. The data collected were assigned a code, and only the principal investigator and collaborators could link the data to the participants. All participants were informed before this study and were asked for verbal consent. This study was approved by the Republic of Kenya Turkana County Government (20 May 2024). 

## 3. Results

**Figure 1:**
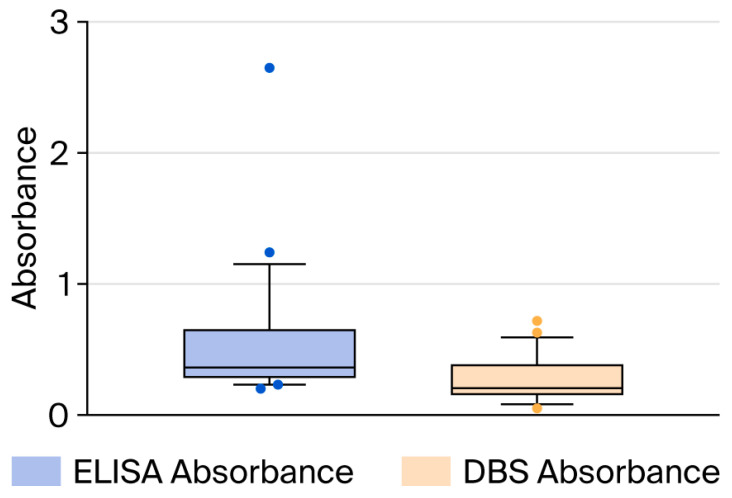
Distribution of absorbance values for the 23 samples obtained via standard ELISA and dried blood spots (DBSs).

**Figure 2:**
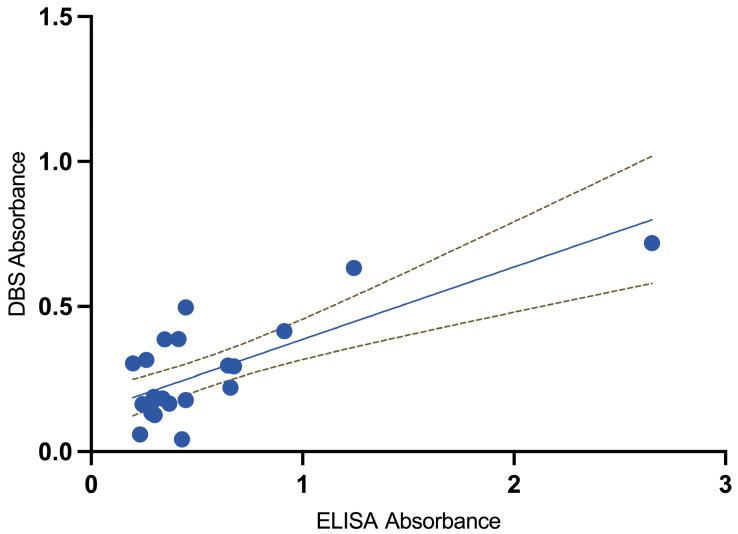
Scatterplot illustrating the relationship between serum ELISA and dried blot spot (DBS) eluate absorbance, along with its 95% confidence interval, for the 23 samples.

We included 15 seropositive and eight seronegative samples for DBS validation and measured *Schistosoma*-specific IgG using ELISA in paired serum samples and DBS eluates. The median serum ELISA absorbance was 0.35 (IQR 0.28–0.65), with a median OD index of 1.19 (IQR 1.00–1.72). In DBS eluates, the median absorbance was 0.22 (IQR 0.16–0.39), and the median OD index was 0.85 (IQR 0.61–1.49). Overall, DBSs produced lower values than serum ELISA, with a median absorbance difference of 0.23 (*p* = 0.056) and a median OD index difference of 0.74 (*p* = 0.12) (Figure 1 and Figure 2).

**Figure 3:**
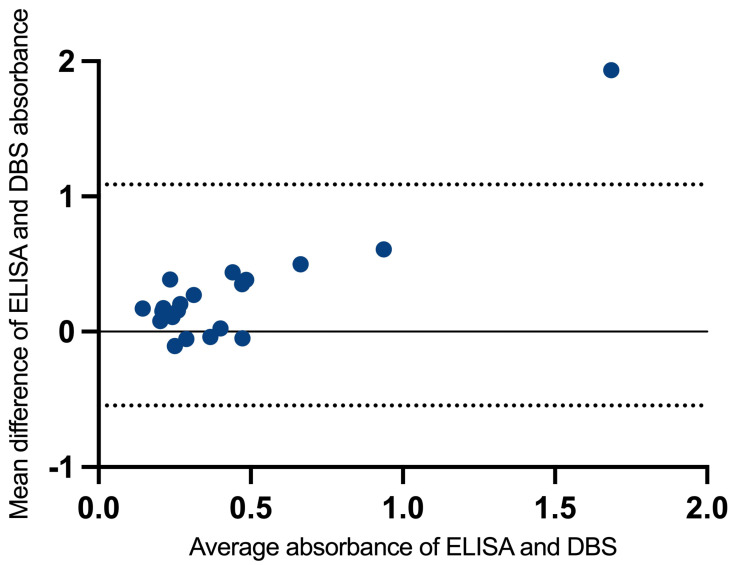
Bland–Altman plot showing agreement between serum ELISA and DBS-derived absorbance measurements, with 95% confidence interval, for the 23 samples (y = 0 indicates perfect average agreement).

Bland–Altman analyses (Figure 3) showed that the differences were centred around a mean bias of 0.27 units (ELISA higher on average), with 95% limits of agreement from −0.54 to 1.01, reflecting the expected variability when comparing two quantitative readouts obtained with different sampling and processing approaches. Overall, these findings support that ELISA and DBS measurements capture a shared underlying signal, with moderate concordance across individuals, while acknowledging some dispersion between paired measurements.

The agreement between the ELISA OD index (used in routine clinical care) and the DBS-derived OD index was evaluated using 23 paired samples. The two methods exhibited a moderate positive correlation (Pearson’s *r* = 0.54; *p* = 0.008), suggesting that participants with higher values in one method generally had higher values in the other. Using Lin’s concordance correlation coefficient, which accounts for both correlation and agreement with the line of equality, the overall concordance was ρc = 0.31 (95% CI 0.104–0.522; *p* = 0.003), indicating a measurable and statistically significant agreement between the methods.

Given the low values observed with the DBS ELISA OD index, we chose a cut-off of >0.8 to enhance sensitivity, positioning the test towards the “upper-left” region of the ROC curve at the expense of reduced specificity. With this threshold and serum ELISA as the reference, DBSs accurately identified 12 out of 15 positives (sensitivity 80%) and 4 out of 8 negatives (specificity 50%), resulting in three false negatives and four false positives. Overall, the discriminatory performance was modest, with an AUC of 0.65 (95% CI 0.44–0.86), indicating limited separation between positive and negative samples and some uncertainty, which is consistent with the small validation sample.

**Figure 4:**
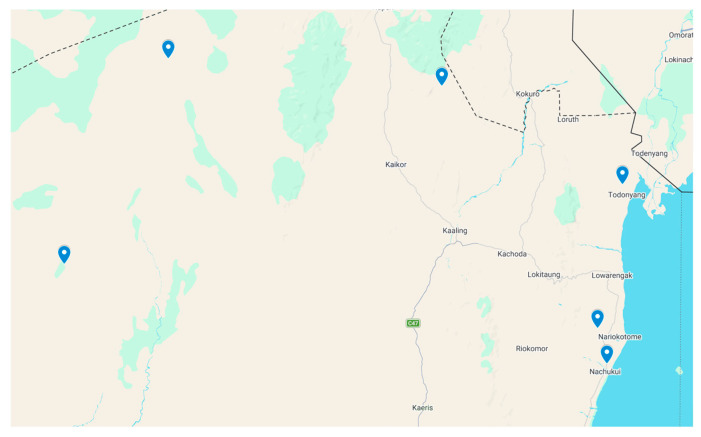
Map showing the sampled communities along the western/northern Turkana County, Kenya (This figure made with Google maps).

Regarding the field study in Turkana, we recruited 155 participants from six locations (Figure 4 and Table 1). Most of them were men (60%) with a median age of 30. Reported exposure to freshwater was almost universal: 154 out of 155 (99.4%) participants reported bathing in ponds, dams, Lake Turkana, or rivers, with only one participant reporting no freshwater contact. The sex distribution varied by site, with a male predominance in most locations, while Lobur included a higher proportion of women (25 out of 38).

The median ELISA OD index for DBS samples was 0.55 (IQR 0.34–0.79). Six cases had results slightly above >0.8, but all of them showed negative IHA results (<1/80), and therefore were not considered true positive results.

## 4. Discussion

In this exploratory serosurvey of adults living in communities in the western region of Lake Turkana, we found no serological evidence of schistosomiasis when using a two-test strategy (ELISA plus indirect haemagglutination) on DBS-derived samples, despite nearly universal self-reported freshwater exposure. In parallel, our DBS validation study against routine serum ELISA showed that DBSs eluates systematically yielded lower quantitative readouts and modest discriminatory performance, favouring a cut-off (>0.8) to increase sensitivity, with moderate correlation and an AUC of 0.65. These results suggest that, in this setting, population-level transmission (or at least cumulative exposure sufficient to generate detectable IgG responses by the DBS approach used) is likely very low or nonexistent, and that DBS-based serology requires careful calibration and context-specific interpretation.

The main strength of our study is its practical, field-oriented design. DBS sampling enabled recruitment across various remote communities and supported centralised testing at a reference laboratory, thereby improving feasibility of surveillance in settings with limited venipuncture, cold-chain, and transport infrastructure. We also aimed to reduce confounding from imported exposure by excluding individuals with extended residence in known endemic areas elsewhere in Kenya. Additionally, we complemented the serosurvey with a study validating DBS performance. 

Some limitations must be acknowledged. The serosurvey relied on antibody detection, which does not distinguish between current and past infections, may miss very recent exposure before seroconversion, and may remain positive for a long time after cure. Our study was based on a non-random sample, and the results should be interpreted as an estimate for the studied population rather than as a fully representative, population-based prevalence measure of the entire region. In addition, malacological surveys would have strengthened the ecological characterisation of transmission. Nevertheless, in this study, our primary aim was to identify whether *Schistosoma* exposure was present in the area by detecting specific antibodies, rather than to characterise the infection type or stage. Second, the DBS validation sample was small (23 paired samples), resulting in leading to imprecise estimates and limiting subgroup analyses by antibody level or clinical phenotype. Additionally, DBS eluates showed a systematic downward bias compared to serum (mean bias 0.27 absorbance units), increasing the risk of false negatives if serum-derived thresholds are used without adjustment. We addressed this by applying a lower ELISA OD index threshold (>0.8), instead of the standard cut-off (>1.1), to classify samples as positive, thus prioritising sensitivity. Similar sensitivity-oriented thresholds have been used in field-based studies of other infections to improve case detection under operational constraints. [20,21,22,23,24]. Finally, our survey population consisted of adults. If local transmission mainly affects school-aged children (as in many endemic areas), adult seronegativity might underestimate ongoing transmission among younger groups.

Schistosomiasis prevalence in Kenya is highly heterogeneous, with well-described high-burden areas (e.g., the Lake Victoria basin and coastal/Tana River settings) and lower prevalence in other locations, and it is shaped by ecological suitability for intermediate host snails, water-contact patterns, and the effects of mass drug administration [10,11,12,13]. Against this background, our finding of no confirmed seropositivity in western Lake Turkana aligns with a scenario of low or absent sustained transmission within the sampled communities, at least during the study period. This result may assist in directing control efforts: it supports prioritising resources and preventive chemotherapy toward established high-burden foci while avoiding unnecessary large-scale interventions in areas where the expected yield is limited. Additionally, it offers locally generated evidence to enhance risk assessment and mapping strategies for future surveillance. Furthermore, our findings help contextualise earlier expatriate seropositivity detected at our centre, which may be more plausibly explained by previous inadvertent exposure outside the Lobur dam area. 

Regarding DBS performance, prior research supports DBSs as a practical approach for serosurveys in mobile or resource-limited settings, but published experience also shows that extraction protocols, storage conditions, and assay-specific cut-offs significantly influence sensitivity and specificity. Our data provide field-relevant parameters to aid in cut-off calibration and the development of confirmatory testing algorithms. Using a conservative threshold may miss low-intensity infections, whereas a more sensitive threshold can increase false positives and overestimate exposure unless results are verified. In this context, a sequential two-test strategy is likely to enhance overall specificity and improve the interpretability of serosurvey results. Operationally, DBSs may be suitable for initial screening and geographical reconnaissance, but positive or borderline results should prompt confirmatory testing (preferably serum-based serology and/or parasitological/antigen detection where feasible), especially when community-level intervention decisions are at stake. Larger validation studies are necessary to evaluate the DBS-based detection of *Schistosoma*-specific antibodies, better understand variability and agreement with conventional serum serology, and define its utility for field surveys. We have an ongoing study at our centre collecting paired DBS and serum samples from over 100 participants.

In summary, our study suggests low detectable or nonexistent schistosomiasis exposure among adults in the surveyed western/northern Lake Turkana communities. It also shows that DBS-based serology is feasible as a screening tool for field studies, although it exhibits modest accuracy without careful calibration and further studies are needed to evaluate and calibrate this technique.

## Figures and Tables

**Table 1:** Baseline characteristics of participants.

	Anam (*n* = 32)	Kaito (*n* = 23)	Loropio (*n* = 22)	Todonyang (*n* = 33)	Lobur (*n* = 38)	Other * (*n* = 7)	Total (*n* = 155)
Sex	M: 24 F: 8	M: 17 F: 6	M: 18 F: 4	M: 19 F: 14	M: 13 F: 25	M: 2 F: 5	M: 93 F: 62
Median age (P25–75)	34 (23–51)	22 (19–29)	29 (24–39)	34 (24–42)	29 (22–39)	31 (20–39)	30 (22–40)
Reported baths in freshwater (Lake Turkana, ponds, dams and rivers)	Y:32 N: 0	Y: 23 N: 0	Y: 22 N: 0	Y: 33 N: 0	Y: 38 N: 0	Y: 6 N: 1	Y: 154 N: 1

F: female; M: male. * Other: Kokuselei (3), Nariokotome (4).

## Data Availability

The raw data supporting the conclusions of this article will be made available by the authors upon request.
